# OLDER ADULT ENGAGEMENT IN COMMUNITY GERONTOLOGY

**DOI:** 10.1093/geroni/igac059.1646

**Published:** 2022-12-20

**Authors:** Natalie Pope

## Abstract

Community gerontology integrates scholarship on aging and communities through the lens of mesolevel environments, the diverse continua of settings linking micro and macro contexts of aging. This interdisciplinary symposium brings together research exploring the interconnections of older adults’ engagement across and within the socio-ecological levels of their lived experiences, place-based communities, and policy contexts. Yeh et al.’s paper elucidates the promises of visual methods in critical qualitative research with older adults to subvert power dynamics and advance social justice in gerontology. Latham-Mintus’ paper explores how “weak ties” created by the interaction of social infrastructure within geospatial places and spaces can facilitate longer, healthier lives. Reyes’ paper uses an intersectional life course perspective to explore placemaking as civic participation among Latinx immigrants and African American older adults across time and social environments. Plasencia’s paper focuses on aging in community among diverse older adults using a photovoice to assess their perceptions and needs within the lived urban environment. Pope and colleagues explore how a multi-state sample of older adults perceive their roles in age-friendly initiatives at individual, organizational, and community levels. Taken together, the papers discuss and contextualize the multidirectional ways older adults engage their place-based communities within larger macro sociopolitical structures – an analysis that highlights the embeddedness of mesolevel community environments both within and between micro and macro spheres. This session further speaks to the importance of centering older adults’ experiences in community gerontology by illuminating the ways older adults not only age in community but through layers of community.

